# Bee-Derived Products in Aquaculture Nutrition: A Comprehensive Review of Impacts on Fish Performance, Health, and Product Quality

**DOI:** 10.3390/ani15213153

**Published:** 2025-10-30

**Authors:** Vittorio Lo Presti, Mauro Cavallaro, Ambra Rita Di Rosa

**Affiliations:** Veterinary Sciences Department, University of Messina, Viale Palatucci, 98168 Messina, Italy; mauro.cavallaro@unime.it (M.C.); ambra.dirosa@unime.it (A.R.D.R.)

**Keywords:** bee-derived products, fish health, aquaculture nutrition

## Abstract

**Simple Summary:**

Aquaculture faces major challenges related to intensive farming, disease outbreaks and environmental stressors that compromise fish health and product quality. Bee-derived products such as pollen, bee bread, propolis, royal jelly, honey and fermented derivatives are rich in bioactive compounds with antioxidant, antimicrobial and immunostimulant properties. Recent studies in species like Nile tilapia (*Oreochromis niloticus*), rainbow trout (*Oncorhynchus mykiss*), European sea bass (*Dicentrarchus labrax*), meagre (*Argyrosomus regius*), and African catfish (*Clarias gariepinus*) demonstrate that dietary inclusion of these natural supplements can enhance growth, immune responses, stress tolerance and even the nutritional profile of fish flesh. For example, bee pollen at 10–30 g kg^−1^ increased growth by ~45% in African catfish, and a 25 g kg^−1^ pollen diet yielded up to 93% protection of Nile tilapia against *Aeromonas hydrophila* infection; in trout, pollen-derived carotenoids increased fillet carotenoid content (≈0.50–0.60 vs. 0.31 mg β-carotene eq kg^−1^ in controls), albeit with lower pigmenting efficacy than synthetic astaxanthin. Nevertheless, results are still fragmented and vary depending on product type, dose and origin. By reviewing current evidence and highlighting critical gaps, this work explores how bee products may support nutritional strategies that improve fish resilience, disease resistance and overall sustainability in aquaculture systems.

**Abstract:**

Aquaculture is expanding rapidly worldwide, but its sustainability is threatened by intensive production practices, environmental stressors and recurrent disease outbreaks. Natural feed additives are increasingly studied as alternatives to antibiotics and synthetic compounds. Among them, bee-derived products—pollen, bee bread, propolis, royal jelly, honey and fermented derivatives—represent a promising resource due to their richness in proteins, amino acids, fatty acids, vitamins, flavonoids and phenolic compounds with demonstrated antioxidant, antimicrobial and immunostimulant properties. Evidence from studies on species such as Nile tilapia, rainbow trout, European sea bass, meagre and African catfish indicates that dietary supplementation with bee products can improve growth performance, immune and antioxidant responses, stress tolerance and resistance to bacterial infections while, in some cases, enhancing the nutritional value and shelf-life of fish products. Prominent examples include ~45% higher growth in African catfish with 10–30 g kg^−1^ bee pollen, up to 93% protection in Nile tilapia fed 25 g kg^−1^ pollen against *Aeromonas hydrophila*, and increased trout fillet carotenoids with pollen-derived pigments (with overall growth unchanged and pigmentation lower than synthetic astaxanthin). Conversely, meagre fed 20–40 g kg^−1^ raw pollen showed reduced growth and digestibility with elevated intestinal stress markers, underscoring species- and dose-specific responses. Nevertheless, the available data remain fragmented and heterogeneous, reflecting differences in product type, origin, dosage and experimental design. This review critically analyses the current knowledge on bee products in aquaculture nutrition, identifies the main gaps and limitations, and outlines future research directions. By linking fish physiology, nutritional strategies and product quality, bee-derived products emerge as innovative tools for promoting fish health and resilience in sustainable aquaculture.

## 1. Introduction

In recent decades, aquaculture has established itself as the fastest-growing animal food production sector worldwide, now providing nearly half of the fish destined for human consumption [1]. This sector represents not only a strategic source of high-quality and easily digestible proteins but also an important vehicle of polyunsaturated fatty acids, vitamins, and essential minerals for human health. Beyond its nutritional value, aquaculture plays a crucial role in global food security, as it reduces pressure on wild fish stocks and ensures a more predictable and sustainable production compared to traditional fisheries.

The rapid expansion of the sector, however, is accompanied by significant challenges. Intensive systems, characterised by high stocking densities and increasingly demanding production practices, expose fish to numerous stress factors, including fluctuations in temperature and water quality, environmental contaminants, and pathogenic agents [1,2]. These conditions compromise physiological and immune resilience, with negative consequences on mortality, growth, and product quality. The prophylactic use of antibiotics and chemotherapeutics, while historically effective in limiting disease outbreaks, has raised increasing concerns about antimicrobial resistance, the accumulation of chemical residues in aquatic ecosystems, and potential risks for consumer health [3]. For these reasons, the search for natural, eco-friendly nutritional strategies has become a priority in modern aquaculture.

The search for natural, eco-friendly nutritional strategies has become a priority in modern aquaculture. In this context, a wide range of natural additives—such as plant extracts, essential oils, probiotics, and other bioactive compounds—has been investigated as alternatives to antibiotics and synthetic chemicals. Within this broader category, bee-derived products have attracted growing attention as potential functional additives for aquaculture.

**Figure 1:**
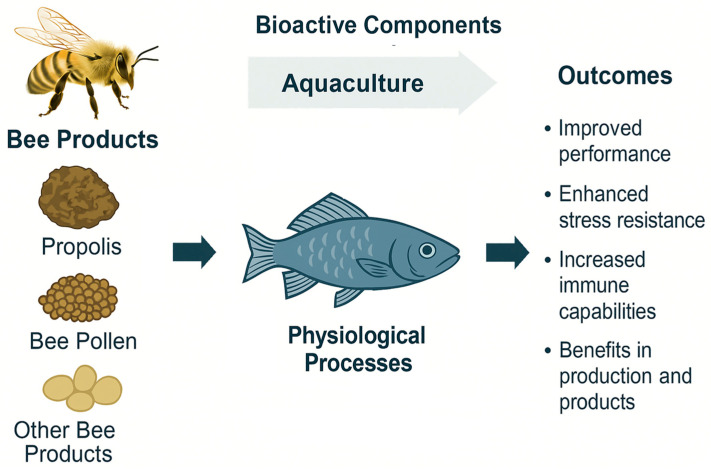
Conceptual overview of the role of bee-derived products in aquaculture. Propolis, bee pollen, and other apicultural products provide bioactive components that interact with fish physiological processes, enhancing growth performance, stress resilience, and immune competence. These interactions ultimately lead to improved production efficiency and product quality in sustainable aquaculture systems.

As summarised in Figure 1, bee-derived products contain a wide range of bioactive compounds that can modulate fish physiological processes, ultimately leading to improved growth, immune function, and stress resilience. This conceptual framework underpins the structure and rationale of the present review.

Bee-derived products—propolis, bee pollen, bee bread, royal jelly, honey, and bee venom—are natural matrices of remarkable complexity, whose chemical composition reflects both botanical origin and environmental conditions. These products contain flavonoids, phenolic acids, amino acids, peptides, fatty acids, vitamins, and enzymes that provide a wide spectrum of biological properties, including antimicrobial, antioxidant, anti-inflammatory, and immunomodulatory activities [4,5,6,7,8]. Beyond their chemical profile, the naturally associated microbiota also plays an important role, as bee pollen and bee bread harbour lactic acid bacteria and other beneficial microorganisms that enhance their probiotic and preservative functions [9,10,11,12].

From a zootechnical perspective, several studies have demonstrated that propolis supplementation can improve growth, innate immunity, and survival in tilapia, trout, and other farmed species. For example, in rainbow trout, dietary propolis increased survival by more than 20% following *Aeromonas hydrophila* infection [13,14,15], although dose-dependent responses have been reported, with high inclusion levels impairing growth performance [16]. In rainbow trout, long-term administration of propolis was safe and well tolerated, without significant alterations in blood biochemical parameters [17], while short-term pollen supplementation enhanced growth and immune responses [18].

Bee pollen has demonstrated beneficial effects across various fish species, generally enhancing growth performance and antioxidant responses. Positive outcomes have been observed in tilapia, where supplementation improved growth and disease resistance [19,20], and in African catfish, where it promoted favourable gut microbiota and nutrient utilisation [21]. Nonetheless, adverse effects such as reduced growth and intestinal stress have been reported in some marine species like meagre, underlining the need for species-specific evaluation before practical application [22].

Although propolis and pollen are the most extensively studied, other bee-derived products deserve attention. Bee bread, due to its microbial fermentation, may exert probiotic and immunomodulatory effects [12]. Royal jelly contains major royal jelly proteins (MRJPs) and fatty acids such as 10-HDA, known for antioxidant and hypolipidemic activities. Honey, while rarely used in aquafeeds, exhibits antimicrobial and nutraceutical properties [5]. Finally, bee venom, rich in peptides such as melittin and apamin, has been scarcely explored in fish, but its well-documented immunomodulatory and cytotoxic properties suggest potential future applications.

In addition to their impact on fish health and farming performance, bee products may also improve the quality of aquaculture products. Their bioactive compounds modulate lipid metabolism and antioxidant defences in muscle tissues, favouring greater oxidative stability and a more favourable nutritional profile, for instance, through improved polyunsaturated fatty acid content. Moreover, the incorporation of bee products in natural coatings has been proposed as a strategy to delay oxidative processes and microbial growth in fish products, with potential to extend shelf-life and increase commercial value [23,24]. In this sense, the application of bee products in aquaculture fits into a “feed-to-fork” approach, with positive implications not only for farming efficiency but also for consumer perception of quality and safety.

Despite these promising findings, significant knowledge gaps remain. Current research is still highly fragmented and disproportionately centred on propolis, as highlighted by the review of de la Cruz-Cervantes et al. [25]. A detailed quantification of this imbalance across bee-derived product categories is provided in Section 2. In contrast, other bee-derived products such as bee bread, royal jelly, honey, and bee venom have received very limited or no attention in aquaculture, leaving their potential largely unexplored. Furthermore, the strong variability in chemical composition—driven by botanical origin, seasonality, and extraction methods—makes direct comparisons between studies problematic. The lack of standardised protocols for dosage, administration, and experimental design further complicates the interpretation of results and hinders the formulation of evidence-based recommendations. Addressing these limitations is essential to unlock the full potential of bee products in aquaculture.

The aim of this review is therefore to provide an updated and critical synthesis of the available experimental evidence on the use of bee-derived products in aquaculture, with particular emphasis on species-specific responses, inclusion levels, and their effects on growth, immunity, stress resilience, and product quality. Beyond summarising the current findings, the review seeks to identify major gaps, highlight methodological limitations, and propose future research priorities. Ultimately, the purpose is to offer a comprehensive scientific basis for the sustainable and evidence-based integration of bee products into aquaculture practices.

To complement this overview, Table 1 provides a comparative summary of the main bee-derived products tested in aquaculture, indicating the species, life stage, dosage, duration, and biological responses observed.

## 2. Methods

This review was conducted as a narrative synthesis of the available literature on the use of bee-derived products in aquaculture. A structured bibliographic search was performed between January and August 2025 in Scopus, Web of Science, PubMed, and Google Scholar, using combinations of the terms “bee products”, “propolis”, “bee pollen”, “bee bread”, “royal jelly”, “honey”, and “bee venom” with “fish”, “aquaculture”, “tilapia”, “trout”, “catfish”, “meagre”, “sea bass”, “sea bream”, “crustaceans”, “shrimp”, “immune response”, “growth”, “oxidative stress”, and “product quality”.

The search retrieved about 200 publications. After removing duplicates and screening, 51 articles were selected as directly relevant, based on the following inclusion criteria: (i) peer-reviewed publications in English; (ii) experimental studies on fish or crustaceans supplemented with bee-derived products; (iii) outcomes related to growth, physiology, immunity, oxidative stress, or product quality. Reviews and analytical studies on the composition and bioactivity of bee products were also considered to provide contextual background. The final reference set covers the period 2005–2025.

Among these 51 publications, approximately 25% focused on propolis, 22% on bee pollen, 12% on honey, 6% on royal jelly, and fewer than 4% on drone brood. Bee bread did not appear as a single-product focus, while no studies were found specifically on bee venom or beeswax. The remaining references (about 31%) provided general or multi-product background information.

**Figure 2:**
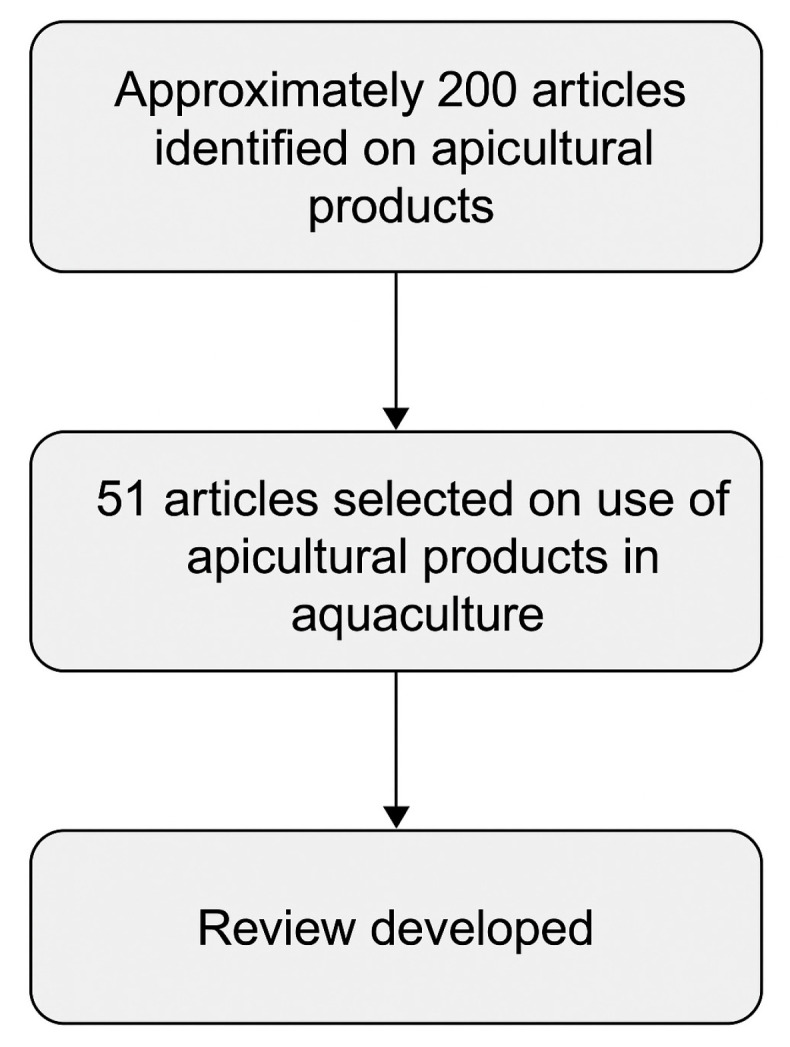
Flowchart summarising the literature search and selection process.

The complete literature identification and screening process is illustrated in Figure 2, which summarises the main steps of the search and selection workflow (identification, screening, eligibility, and inclusion). Despite the inclusion of search terms referring to crustaceans, no specific studies on these species were found, suggesting that this gap reflects the limited availability of data rather than a bias in the search strategy.

## 3. Bee Pollen

### 3.1. Composition and Bioactive Compounds

Bee pollen is a nutrient-dense natural matrix whose composition depends on botanical origin, geographical area, and post-harvest processing. On average, it contains 40–60% carbohydrates, 15–25% proteins, and 1–10% lipids, together with vitamins, minerals, and a wide range of secondary metabolites such as flavonoids and phenolic acids that account for its antioxidant and immunomodulatory potential [4,5].

The outer exine layer, rich in sporopollenin, provides structural protection but also limits nutrient accessibility and digestibility, particularly in carnivorous species such as meagre [22].

Different processing and extraction techniques have been explored to enhance the bioavailability of pollen nutrients and bioactives. Conventional ethanolic and hydroalcoholic extractions increase the recovery of polyphenols and flavonoids, improving the antioxidant capacity of the extract [7,33]. More advanced approaches such as supercritical fluid extraction have been developed to concentrate specific bioactive fractions with higher purity and activity [33].

These compositional and technological aspects define the nutritional and functional value of bee pollen and explain the growing interest in it as a natural additive in aquaculture [18,19,20,21]. This complex mixture of proteins, amino acids, fatty acids, vitamins, and phenolic compounds supports a variety of biological functions that depend on species, trophic level, and processing method [25]. Studies in different fish models have provided preliminary evidence of its capacity to influence metabolic and physiological parameters [34,35,36], confirming that both composition and extraction technology determine its bioactive potential.

Altogether, bee pollen represents not only a rich nutrient source but also a reservoir of bioactive molecules whose specific roles are discussed in detail in the following sections.

### 3.2. Experimental Evidence in Fish

**Nile tilapia**

Dietary inclusion of bee pollen at 20–25 g kg^−1^ diet for 10–60 days improved growth, innate immunity, and resistance to *Aeromonas hydrophila* by increasing phagocytic activity and serum lysozyme [19,20]. Lysozyme is a key effector of innate immunity that hydrolyses bacterial cell wall peptidoglycans, while enhanced phagocytic activity indicates activation of macrophages and neutrophils, reinforcing the first line of host defence. Under pesticide-induced stress, supplementation with 25 g kg^−1^ diet pollen normalised for 30 days antioxidant enzymes (SOD, CAT, GPx), reduced malondialdehyde (MDA), restored biochemical variables, and alleviated histopathological damage in liver and gills [37]. These outcomes demonstrate that phenolic and flavonoid compounds from pollen can mitigate oxidative stress by stimulating enzymatic scavengers of superoxide radicals and hydrogen peroxide, thereby protecting hepatocytes and gill epithelial cells from lipid peroxidation and necrosis.

**Rainbow trout**

Chestnut bee pollen included at 10–40 g kg^−1^ diet for 60 days increased serum immunoglobulins and antioxidant enzyme activities, indicating improved innate immunity and oxidative balance [7,18]. In addition to growth enhancement, hydroalcoholic extracts contributed to better fillet quality and reduced markers of physiological stress. The increase in immunoglobulins, particularly IgM, reflects stimulation of B lymphocytes and enhanced adaptive humoral immunity, which is crucial in aquaculture conditions where fish are constantly challenged by opportunistic pathogens. The upregulation of antioxidant enzymes further indicates that pollen bioactives bolster systemic redox homeostasis, improving resilience to environmental or handling stress.

Using bee pollen as a natural carotenoid source, carotenoids were extracted in sunflower oil (pigmented vegetable oil, PVO) and top-coated onto pellets at 25 or 50 mg total carotenoids kg^−1^ (P1, P2) for 60 days. No mortality occurred and total growth over the full period did not differ among groups; however, the higher carotenoid level (P2) yielded transient improvements in periodical weight gain and specific growth rate. Notably, the viscerosomatic index was lowest in P1 (8.80 ± 0.31%). Instrumental colour showed that synthetic astaxanthin (50 mg/kg; C+) produced the highest redness (a) and SalmoFan^TM^ (DSM, Kaiseraugst, Switzerland) score (25.7), whereas P1–P2 achieved intermediate pigmentation (SalmoFan^TM^ ≈ 20–21) and significantly higher fillet carotenoid content than the negative control (TCC, mg β-carotene eq/kg fillet: C− 0.31; P1 0.50; P2 0.60; C+ 1.62). These data support bee pollen–derived carotenoids as a feasible, solvent-free alternative to promote salmonid flesh coloration, albeit with lower pigmenting efficacy than synthetic astaxanthin under the tested inclusion levels [26].

**Gilthead seabream (*Sparus aurata*)**

The inclusion of 5–10 g kg^−1^ diet supercritical fluid extract of bee pollen for 60 days enhanced peroxidase, protease and bactericidal activities, as well as antioxidant enzyme activity, without compromising growth [33]. Similarly, in gilthead seabream, the 10 g kg^−1^ diet SFE bee pollen diet significantly up-regulated hepatic *il-1β*, *il-6*, and *il-8* expression, while crude pollen depressed *il-8*, confirming that extraction methods can unlock the functional potential of pollen by increasing the bioavailability of phenolic compounds and modulating immune pathways in a dose- and form-dependent manner [34].

**Meagre**

Juveniles fed 20–40 g kg^−1^ diet of raw pollen for 90 days displayed reduced growth and digestibility, intestinal histological alterations, and upregulated TNF-α and HSP70, markers of inflammatory and cellular stress responses [22]. Elevated TNF-α indicates stimulation of pro-inflammatory cytokine pathways, while HSP70 acts as a molecular chaperone upregulated during cellular stress to prevent protein misfolding. The concurrent increase in these markers suggests that the indigestible sporopollenin wall of pollen grains may trigger intestinal irritation and stress, compromising nutrient absorption and growth. This highlights a species-specific limitation, where carnivorous fish with less flexible digestive physiology cannot efficiently utilise raw pollen.

**African catfish**

Bee pollen supplementation (10–30 g kg^−1^ diet for 21 days) significantly improved growth performance (+45% vs. control), villus morphology, and beneficial lactic acid bacteria in the gut microbiota, while maintaining normal liver histology [21]. Enhanced villus length and surface area provide a direct anatomical basis for improved nutrient assimilation, explaining the observed growth benefits. The enrichment of lactic acid bacteria indicates prebiotic activity of pollen polysaccharides, which foster symbiotic microbiota beneficial for gut health and immune modulation. The preservation of hepatic architecture further demonstrates the safety of pollen at tested levels, with no hepatotoxic effects.

**Zebrafish (*Danio rerio*)**

Feeding breeders with pollen—ground bee pollen administered at approximately 3% of body weight per meal, up to two meals per day, for a total feeding duration of 60 days-enhanced larval survival after viral (SVCV) and bacterial (*Salmonella*) challenges, suggesting vertical immunity transfer [35]. Vertical transmission of immunity likely involves deposition of maternal immunoglobulins, antimicrobial peptides, or other immune factors into eggs, thereby increasing offspring resistance to pathogens Other studies, involving the administration of bee pollen powder at 10–30 g kg^−1^ diet for a period of 30–60 days, revealed a profound remodelling of the intestinal microbiota (↓ Aeromonas, ↑ Bifidobacterium breve) and, unexpectedly, an acceleration of tumour growth in melanoma transplant models [36]. Microbiota shifts suggest a probiotic-like action of pollen, promoting beneficial taxa that enhance gut health, while the tumour-promoting effect highlights potential risks of immune overstimulation or altered signalling pathways. This duality underscores both the promise and the complexity of pollen bioactivity in experimental models.

To provide a comparative overview of the experimental evidence, Table 2 summarises the main studies on bee pollen supplementation in fish species. The table reports the inclusion level, trial duration, physiological parameters assessed, and key outcomes. Collectively, the studies highlight the species-specific and dose-dependent nature of bee pollen effects, ranging from improved growth, immunity and oxidative stress responses in tilapia, catfish and trout, to adverse effects in meagre when high dietary levels are used, and novel insights from zebrafish models on vertical immunity transfer and gut microbiota modulation.

### 3.3. Effects on Growth, Immunity and Stress Response

**Figure 3:**
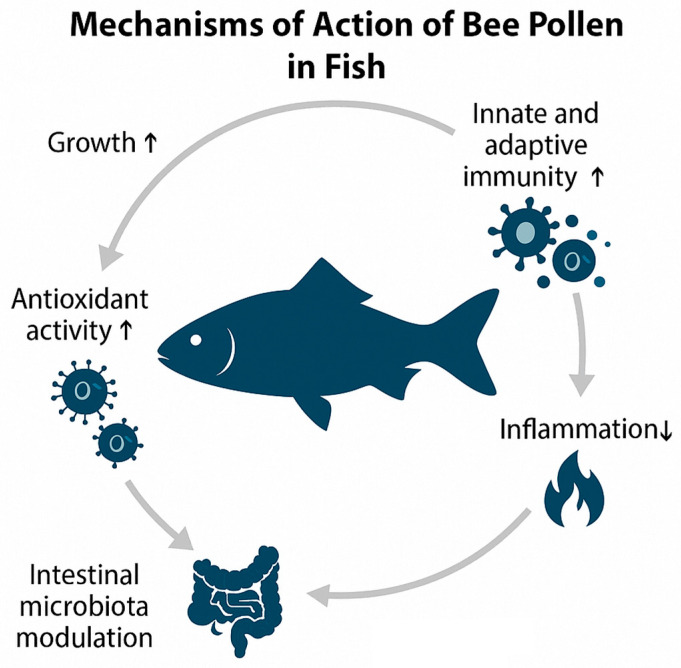
Mechanisms of action of bee pollen in fish. The diagram illustrates the main physiological effects of dietary bee pollen, including enhanced antioxidant activity, modulation of intestinal microbiota, stimulation of innate and adaptive immunity, reduced inflammation, and overall improvement in growth performance. ↑ increase; ↓ decrease.

The effects of bee pollen on fish performance and health are strongly species- and dose-dependent, reflecting differences in digestive physiology, metabolic plasticity, and immune system responsiveness. These physiological mechanisms are summarised in Figure 3, which illustrates the main pathways through which bee pollen enhances digestion and nutrient absorption, modulates gut microbiota, strengthens antioxidant and immune defences, reduces oxidative and inflammatory stress, and ultimately supports growth and product quality.

**Growth performance and nutrient utilisation.**

In omnivorous species such as Nile tilapia and African catfish, moderate dietary inclusion of 10–30 g kg^−1^ diet bee pollen for 30–60 days consistently enhanced growth and feed efficiency [20,21]. These benefits are associated with improved intestinal villus morphology and expansion of the absorptive surface, which facilitates nutrient uptake, as well as prebiotic modulation of gut microbiota favouring lactic acid bacteria and other beneficial taxa. Synergistic effects have also been reported when bee pollen was combined with probiotic supplementation in tilapia and gilthead seabream, resulting in improved growth performance, modulation of beneficial gut microbiota, and enhanced muscle quality traits [33]. By contrast, in carnivorous species such as meagre, higher dietary levels (up to 40 g kg^−1^ for 75 days) impaired growth and diet digestibility, leading to intestinal histological damage and systemic stress [22]. This outcome reflects the limited ability of carnivorous fish to digest the sporopollenin-rich pollen wall, resulting in mucosal irritation and reduced assimilation of nutrients.

**Innate and adaptive immune responses.**

Immunostimulatory activity of bee pollen has been demonstrated across several species. In tilapia (10–30 g kg^−1^ diet, 30–60 days), increases in lysozyme and phagocytic activity indicate reinforcement of innate defence barriers [20]. In addition, higher peroxidase and protease/antiprotease activities were observed in seabream receiving supercritical fluid extracts (10–40 g kg^−1^ diet, 10–60 days), suggesting improved regulation of inflammation and humoral immunity [18,19]. Rainbow trout trials also showed elevated IgM levels, reflecting stimulation of B lymphocytes and enhancement of adaptive humoral immunity [7,18]. Collectively, these results highlight that bee pollen can strengthen both innate and adaptive components of the immune system, thereby improving resilience against bacterial and viral challenges.

**Antioxidant defences and oxidative stress.**

Bee pollen bioactives, particularly flavonoids and phenolic acids, contribute to reinforcement of antioxidant defences. Supplementation has been associated with upregulation of enzymatic antioxidants (SOD, CAT, GPx) and reduction in lipid peroxidation markers such as MDA in tilapia (25 g kg^−1^ diet, 30 days) and trout (10–30 mL/kg HPE, 60 days) [7,37]. These mechanisms mitigate oxidative stress induced by environmental contaminants or aquaculture stressors, preserving membrane integrity and protecting hepatocytes and gill tissues from damage. Similarly, Ferreira et al. [27] showed that in South American catfish exposed to the fungicide tebuconazole, waterborne administration of pollen (0.01, 0.03 and 0.05 g L^−1^, 4 days) significantly enhanced SOD, CAT and GST activities while reducing lipid peroxidation and protein carbonylation, thereby preventing oxidative damage.

**Inflammatory and stress responses.**

Excessive inclusion (20–40 g kg^−1^ diet, 90 days) of raw pollen may have adverse effects, particularly in carnivorous fish. In meagre, increased expression of *tnf-α* and *hsp70* indicated activation of pro-inflammatory cytokine signalling and cellular stress pathways [22]. *Tnf-α* is a central mediator of inflammatory cascades, while *hsp70* is a molecular chaperone induced under conditions of protein misfolding and oxidative stress. Their elevation provides mechanistic evidence that high pollen levels can trigger intestinal inflammation, impair tissue homeostasis, and compromise performance.

**Reproductive function and transgenerational immunity.**

Zebrafish studies have added novel insights into the potential of bee pollen to modulate reproduction and immunity across generations. Dietary supplementation (3% of body weight, 90 days) of breeders enhanced larval survival following viral and bacterial challenges, consistent with the vertical transfer of immune effectors such as maternal immunoglobulins or antimicrobial peptides [35]. This represents an important physiological mechanism through which dietary components can influence offspring fitness and survival.

**Gut microbiota modulation and risks.**

Pollen supplementation (10–30 g kg^−1^ diet, 30–60 days) has also been shown to reshape gut microbial communities in zebrafish, reducing opportunistic pathogens such as *Aeromonas* and promoting beneficial taxa such as *Bifidobacterium breve* [36]. This prebiotic-like effect supports intestinal health and may synergise with immune enhancement. However, unexpected outcomes have also been reported: in melanoma-bearing zebrafish, pollen supplementation accelerated tumour growth, suggesting possible immune overstimulation or altered inflammatory signalling [36]. These findings highlight both the opportunities and the risks of using bee pollen, emphasising the importance of dose optimisation and species-specific evaluation.

### 3.4. Limitations and Research Needs

Despite the promising evidence on bee pollen as a functional feed additive in aquaculture, several limitations constrain its broader application and call for further research.

**Variability in composition and bioactivity.**

The chemical profile of bee pollen is highly dependent on botanical and geographical origin, seasonality, and processing methods. This variability directly influences its content of proteins, flavonoids, carotenoids, and polyunsaturated fatty acids, which in turn determine its immunomodulatory and antioxidant capacity. As a result, experimental outcomes are difficult to compare across studies, and standardisation of pollen characterisation is urgently needed.

**Digestibility and species-specific responses.**

The sporopollenin-rich pollen wall is poorly digestible, especially in carnivorous fish with shorter and less specialised digestive tracts. This limitation was evident in meagre, where raw pollen supplementation triggered intestinal inflammation and stress responses [22]. Conversely, omnivorous fish such as tilapia and catfish benefitted from improved nutrient assimilation and growth [20,21]. Pre-treatment methods, including micronisation, enzymatic hydrolysis, and supercritical fluid extraction, enhance bioavailability of bioactives and may overcome these species-specific constraints [33].

**Incomplete mechanistic understanding.**

Most studies have assessed general biomarkers (growth, survival, lysozyme, antioxidant enzymes), but the underlying physiological pathways remain insufficiently explored. For instance, the precise mechanisms of vertical immunity transfer observed in zebrafish [35] or the tumour-promoting effects in melanoma-bearing models [36] are not yet clarified. Omics-based approaches, including transcriptomics, proteomics, and metabolomics, could provide deeper insight into the molecular targets of pollen bioactives.

**Potential risks and safety concerns.**

While low-to-moderate inclusion levels improve immunity and oxidative stress resilience, higher doses may cause adverse effects, such as pro-inflammatory activation and cytotoxic stress [22]. Moreover, the tumour growth reported in zebrafish models [36] raises concerns about the long-term safety of pollen as an immunomodulator. Rigorous toxicological assessments and chronic exposure trials are required to define safe inclusion thresholds.

**Research priorities.**

Future studies should focus on:

1. Establishing dose–response curves across multiple fish species with different feeding guilds.
2. Standardising pollen processing and characterisation to ensure reproducible bioactivity.
3. Applying multi-omics platforms to elucidate the molecular mechanisms behind immunomodulation, antioxidant defence, and microbiota interactions.
4. Investigating the impacts of pollen supplementation on product quality traits (lipid composition, oxidative stability, shelf-life).
5. Conducting long-term and large-scale trials under farm conditions to validate laboratory findings and assess economic feasibility.

## 4. Propolis

### 4.1. Composition and Bioactive Compounds

Propolis is a resinous material that honeybees collect from plant buds, exudates, and other vegetal secretions, blending them with wax and salivary enzymes to produce a sticky substance used to seal and protect the hive. The material serves multiple biological functions for the colony, including structural reinforcement, pathogen defence, and maintenance of internal homeostasis. Its chemical composition is highly variable and depends on the botanical and geographical origin of the plants visited by the bees, as well as on seasonality and collection methods. On average, propolis consists of approximately 50% resins and balsams, 30% waxes, 10% essential and aromatic oils, and 5% pollen and other minor constituents [17,38].

To date, more than 300 distinct compounds have been identified in different propolis samples, encompassing flavonoids, phenolic acids and their esters, terpenes, steroids, amino acids, vitamins, and minerals [17]. The qualitative and quantitative profiles of these compounds differ substantially between tropical and temperate regions, and even among apiaries located within a few kilometres of each other, reflecting the high botanical selectivity of bees and the local plant biodiversity.

The polyphenolic fraction—mainly flavonoids such as pinocembrin, galangin, chrysin, and quercetin, together with phenolic acids and their derivatives such as caffeic acid phenethyl ester (CAPE)—is considered the main determinant of the biological properties of propolis [13,14]. These molecules act synergistically as potent antioxidants and antimicrobial agents, contributing to the neutralisation of free radicals, inhibition of lipid peroxidation, and stabilisation of cellular membranes. In addition, they modulate pro-inflammatory pathways by influencing cytokine release and the expression of enzymes involved in oxidative metabolism.

Although the relative abundance of individual constituents varies widely among propolis types (e.g., poplar-type, Baccharis-type, or Dalbergia-type), their combined presence confers a consistent set of biological properties. These include antimicrobial, antioxidant, anti-inflammatory, and immunomodulatory actions, which have been observed not only in mammals but also in aquatic organisms [3,8,13,15,16,17,25]. The chemical complexity of propolis also explains its capacity to interact with metabolic and detoxification processes, thereby protecting tissues from oxidative and xenobiotic stress [26].

Altogether, propolis can be viewed as a complex phytochemical system, rich in resins and phenolics, that functions as a natural reservoir of protective compounds. Its molecular diversity, combined with strong geographical and botanical influences, makes it an exceptionally versatile natural product, whose bioactivity potential and specific effects in aquaculture species are discussed in the following sections.

### 4.2. Experimental Evidence in Fish

**Nile tilapia**

Experimental trials in Oreochromis niloticus have consistently demonstrated that dietary supplementation with crude or ethanolic propolis extracts enhances growth performance, immune function, and resistance to *Aeromonas hydrophila*. Abd-El-Rhman [13] reported that inclusion of ethanolic extract of propolis (1–3 g kg^−1^ diet for 30 days) significantly increased lysozyme activity and serum bactericidal capacity, two major components of the humoral innate immunity. Lysozyme hydrolyses bacterial cell wall peptidoglycans, while serum bactericidal activity reflects activation of complement and antimicrobial peptides, enabling rapid elimination of *Aeromonas hydrophila*. Orsi [16] confirmed these effects using Brazilian crude propolis powder (10–50 g kg^−1^ diet for 30 days), observing changes in neutrophil counts and nitric oxide levels consistent with macrophage activation and respiratory burst. Under bisphenol-A exposure, Hamed [3] demonstrated that propolis inclusion (9 g kg^−1^ diet for 45 days) normalised antioxidant enzymes (SOD, CAT, GPx) and reduced hepatic lipid peroxidation (MDA), thereby protecting hepatocytes and gill epithelia from oxidative stress. Histological analyses revealed preserved liver architecture and reduced necrosis in supplemented groups. Abbass [20] observed synergistic benefits when propolis was combined with bee pollen (9 g kg^−1^ each for 60 days), improving haematological indices, serum proteins, and fecundity. More recently, Ranjel [15] reported that ethanolic extracts of local propolis (10–15 g kg^−1^ diet for 28 days) improved juvenile growth, muscle fibre development, and protein accretion, effects possibly mediated by myostatin-related regulatory pathways.

**Rainbow trout**

Meurer [14] demonstrated quadratic growth responses to brown propolis extract, with maximal weight gain at 2.2 g kg^−1^ of diet after 60 days.

The improvement was attributed to antimicrobial activity in the gut lumen, reducing microbial competition and facilitating nutrient assimilation. Kashkooli [17] confirmed long-term safety: serum biochemistry remained stable across propolis doses up to 9 g kg^−1^ for 60 days, ruling out hepatotoxicity or metabolic disturbance. Collectively, these findings indicate that propolis is safe and can support growth by modulating gut microbiota and digestive physiology.

**Gilthead seabream**

Cuesta [38] reported enhanced phagocytic and cytotoxic activity in leukocytes after in vivo administration of propolis (10 mg fish^−1^,15 days). Increased phagocytosis reflects activation of macrophages and neutrophils, while elevated cytotoxic responses point to stimulation of non-specific cytotoxic cells and NK-like activity. These outcomes demonstrate that propolis reinforces cellular immunity, improving the ability to recognise and eliminate pathogens.

**European sea bass**

Under acute cold stress, Šegvić-Bubić [8] observed that propolis-enriched diets containing propolis extract (1–3 g kg^−1^ of diet for 40 days) improved growth performance and stabilised serum proteins and enzymes. Immunohistochemical markers showed reduced expression of stress proteins, while lower plasma cortisol and glucose levels indicated attenuation of the hypothalamic–pituitary–interrenal (HPI) axis activation.

More recently, a series of studies confirmed and expanded these findings. Islam [39] demonstrated that dietary administration of propolis (≈4.5 g kg^−1^ of diet for 60 days) enhanced growth, survival, red blood cell indices, and innate immune markers while decreasing cortisol levels and hsp70 expression during experimental heatwaves (32 °C). In subsequent studies, the same group reported that propolis, alone or combined with vitamins C and E or phycocyanin (4.5 g kg^−1^ of diet for 60 days), mitigated biochemical markers of hepatic and renal stress, preserved glucose and lipid metabolism, and upregulated growth- and immunity-related genes under both heat and cold stress conditions [40,41].

More recently still, Islam [42] showed that propolis supplementation interacted positively with reduced salinity (12 PSU, 60 days), further enhancing stress resilience, antioxidant capacity, and endocrine responses during high-temperature exposure. Overall, these studies indicate that propolis supplementation in various freshwater and marine fish species promotes growth, feed efficiency, and stress tolerance through the modulation of antioxidant and immune systems and the maintenance of endocrine balance.

Optimal inclusion levels generally range between 1 and 5 g kg^−1^ of diet for 30–60 days, although species-specific differences and the potential adverse effects of higher dosages must be considered. Table 3 summarises the main aquaculture studies on propolis supplementation, reporting for each trial the dose, duration, evaluated parameters, and main outcomes. Overall, the evidence shows that propolis improves performance and immune status in tilapia, trout, and sea bass, and enhances innate immune responses in seabream, though further research is needed to establish safe and standardised application protocols.

### 4.3. Effects on Growth, Immunity and Stress Response

**Figure 4:**
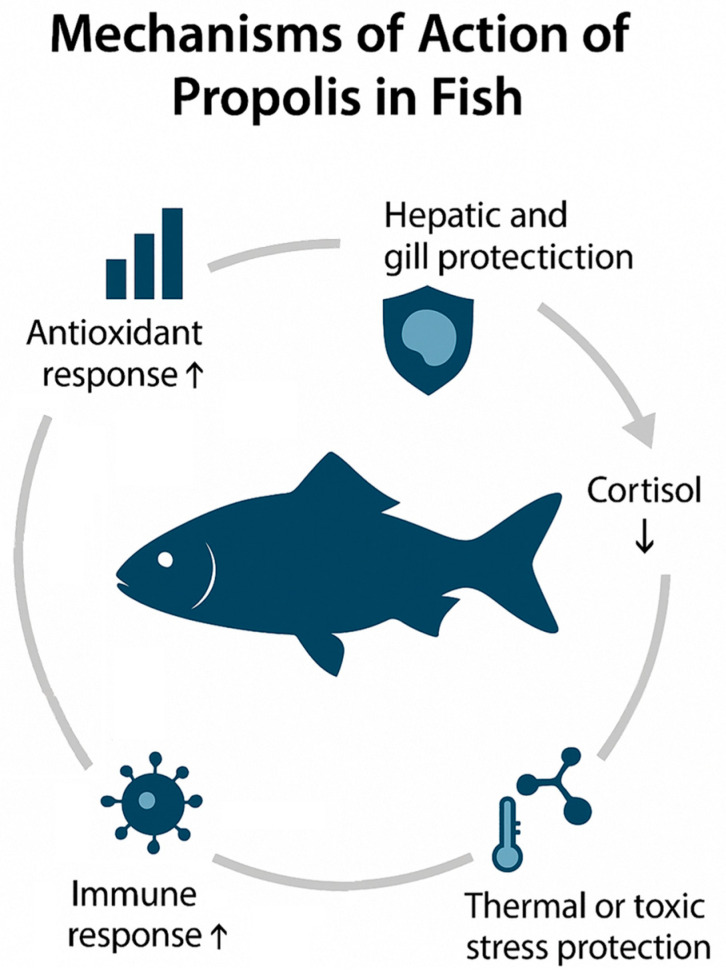
Mechanisms of action of propolis in fish. The diagram illustrates the main physiological pathways through which propolis enhances antioxidant enzyme activity, stimulates immune responses, reduces cortisol and pro-inflammatory mediators (TNF-α, HSP70), protects hepatic and gill tissues, and improves resistance to thermal or toxic stress. ↑ increase; ↓ decrease.

The beneficial effects of propolis on fish physiology are closely related to its polyphenolic composition and depend on both species-specific metabolism and environmental conditions. These effects are mediated through multiple pathways involving antioxidant, immunomodulatory, and anti-inflammatory mechanisms. As summarised in Figure 4, propolis enhances antioxidant enzyme activities (SOD, CAT, GPx), stimulates innate and adaptive immune responses, downregulates pro-inflammatory mediators (TNF-α, IL-1β, HSP70), protects hepatic and gill tissues from oxidative damage, mitigates stress-related cortisol elevation, and overall contributes to improved growth performance and resilience under environmental or metabolic stress.

**Growth performance and nutrient utilisation**.

Ethanolic propolis extracts improved growth and feed efficiency in tilapia and sea bass [8,13,15]. In *Nile tilapia*, dietary inclusion of ethanolic or crude propolis extracts at 1–3 g kg^−1^ diet for 30 days enhanced growth performance, feed conversion ratio, and resistance to *Aeromonas hydrophila* [13,15,16]. In European sea bass, these performance outcomes extended to climate-relevant challenges: under experimental heatwaves, supplementation with 4.5 g kg^−1^ diet for ~60 days improved growth and survival and tempered endocrine and cellular stress responses; under cold exposure, it maintained metabolic homeostasis and supported growth- and lipid-metabolism pathways [39,40,41]. Such context-dependent benefits suggest a role for propolis in climate-smart aquafeeds. Mechanisms include enhanced digestive efficiency, mucosal protection, and stimulation of protein synthesis. Positive outcomes were also reported in *Tilapia post-larvae*, where ethanolic propolis extract at 0–4 g kg^−1^ diet for ~30 days improved growth indices and intestinal histomorphometry [43], and in *Nile tilapia juveniles*, where supplementation with 0.5–1.5 g kg^−1^ diet for 45 days enhanced energy digestibility and villus morphology [43]. Brown propolis extract promoted growth in rainbow trout when administered at 1–3 g kg^−1^ diet for 30 days, likely by reducing gut microbial load [14]. By contrast, crude propolis at high inclusion levels (≥2.5–3 g kg^−1^ diet for 30 days) showed neutral or inconsistent effects, reflecting lower bioavailability of bioactives [17].

**Innate and adaptive immune responses.**

Propolis reinforced both humoral and cellular immunity. In *Nile tilapia*, diets containing ethanolic propolis extract (10 g kg^−1^ diet, 30 days) increased lysozyme activity, serum bactericidal capacity, and nitric-oxide production, key indicators of innate defences [13,16]. In Mozambique tilapia, ethanolic propolis extract supplied at 10–20 g kg^−1^ diet for 60 days enhanced growth and activated innate immune responses, including lysozyme and phagocytosis [44]. In gilthead seabream, dietary propolis (1 g kg^−1^ diet, 30 days) increased phagocytic and cytotoxic activity of leukocytes, stimulating macrophages and NK-like cells [38]. In rainbow trout, supplementation with ethanolic propolis extract (10–20 g kg^−1^ diet for 56 days) promoted favourable leukocyte dynamics [14]. Collectively, these outcomes confirm that propolis activates multiple immune pathways, improving resilience against bacterial pathogens.

**Antioxidant defences and oxidative stress.**

Phenolic acids and flavonoids in propolis upregulate antioxidant enzymes and reduce lipid peroxidation. In *Nile tilapia* exposed to bisphenol-A stress, dietary ethanolic extract at 9 g kg^−1^ diet for 42 days restored SOD, CAT and GPx activities, decreased MDA levels, and preserved hepatocyte and gill integrity [3]. These mechanisms protect cellular membranes and maintain redox balance under pollutant or management stress.

**Inflammatory and stress responses.**

Propolis modulates pro-inflammatory cytokines and stress proteins. In sea bass, supplementation with 4.5 g kg^−1^ diet for 60 days reduced *tnf-α* and *hsp70* expression, and lowered cortisol and glucose, indicating downregulation of the HPI stress axis [8]. Subsequent studies substantiated these effects during thermal extremes, showing lower cortisol and enzyme leakage together with higher *igf1* and selective up-/down-regulation of *hsp70*, *tnf-α* and *glut2* in a tissue-specific fashion [39,41].

In Nile tilapia, diets containing ethanolic propolis extract (10 g kg^−1^ diet, 30 days) enhanced nitric-oxide production, confirming macrophage activation and improved pathogen killing [16]. These findings demonstrate that propolis both stimulates effective immune activation and prevents excessive inflammation or chronic stress.

### 4.4. Limitations and Research Needs

Despite the promising evidence, the use of propolis in aquaculture faces several limitations. One of the main challenges is its high compositional variability, which depends strongly on botanical sources, geographic origin, seasonality, and extraction methods [3,16]. This heterogeneity makes it difficult to compare results across studies and to establish consistent recommendations for practical use. Sánchez [26] emphasised that the chemical variability of propolis remains one of the main bottlenecks to its industrial application, reinforcing the need for standardised extraction methods and dosage protocols.

Furthermore, most available trials are short-term and focus primarily on Nile tilapia [13,15], with far fewer data available for salmonids such as rainbow trout [17], marine fish of commercial importance such as seabass and seabream [8,38], and far fewer data available for salmonids such as rainbow trout and marine fish of commercial importance such as seabass and seabream.

More recently, a series of controlled trials on European sea bass demonstrated that dietary propolis at moderate inclusion levels 4.5 g kg^−1^ diet can mitigate abiotic stressors such as acute cold, experimental heatwaves, and salinity fluctuations, improving survival, stabilising biochemical and haematological parameters, and modulating the expression of growth and immunity-related genes [39,40,41,42]. While these findings are encouraging and highlight the potential role of propolis in enhancing resilience to climate-related stressors in marine aquaculture, they all derive from a single research line, and no comparable evidence is yet available for other bee products or different marine species.

Another important limitation is the lack of standardised protocols regarding dosage, duration of administration, and form of supplementation. While ethanolic extracts are widely used due to their enhanced bioactivity [3,15], comparisons with crude propolis are scarce, and dose–response relationships remain poorly defined. The observation that high dietary inclusion can impair growth performance and alter haematological parameters [16] highlights the need for careful calibration of supplementation levels.

Future research should prioritise long-term, multi-species trials supported by thorough chemical characterisation of the propolis used, ideally combining chromatographic profiling and metabolomic analyses to link specific compounds with biological outcomes. Moreover, studies assessing the impact of propolis supplementation on aquaculture product quality, particularly lipid composition, oxidative stability, and shelf-life, remain largely unexplored. Addressing these gaps will be essential to unlock the full potential of propolis as a functional additive for sustainable aquaculture. Recent analyses have also emphasised that antioxidant and immunostimulatory effects of propolis can vary widely depending on botanical origin and extraction methods, reinforcing the need for product standardisation before large-scale application in aquaculture [45].

## 5. Other Bee-Derived Products

Besides pollen and propolis, honeybees produce several other products—namely bee bread, royal jelly, honey, and beeswax—that are of considerable interest in human nutrition and health. However, their use in aquaculture has been far less explored, with only fragmentary evidence available. For this reason, these products are discussed together in a single section. By presenting them collectively, we aim to summarise their unique compositional traits, review the scarce experimental findings, and highlight both their potential applications and the critical gaps that still limit their practical use in aquaculture.

### 5.1. Composition and Bioactive Compounds

The biochemical composition of the less-studied bee products reflects their diverse biological origins within the hive and accounts for their potential as functional ingredients in aquaculture.

**Bee bread** is the result of pollen fermentation inside the hive, where lactic acid bacteria and yeasts convert stored pollen into a more stable and digestible substrate. This fermentation process increases the concentration of free amino acids, vitamins, and short-chain fatty acids, while reducing the content of anti-nutritional factors such as sporopollenin. As a consequence, bee bread is more digestible than raw pollen and contains microbial metabolites that may exert probiotic effects, together with enhanced antioxidant activity [4,23].

**Royal jelly** is a glandular secretion of nurse bees, produced to feed larvae and the queen. It is particularly rich in proteins, with major royal jelly proteins (MRJPs) representing more than 80% of the soluble fraction. Unique lipids such as 10-hydroxy-2-decenoic acid (10-HDA) contribute to its well-known antimicrobial and immunomodulatory activities, while vitamins, minerals, acetylcholine, and bioactive peptides add further functional value [23,25]. Royal jelly is therefore considered one of the most bioactive and nutritionally dense hive products, although its limited availability and high cost restrict its use.

**Honey** is primarily composed of simple sugars, with fructose and glucose representing up to 75% of its dry matter. In addition, honey contains disaccharides and oligosaccharides, organic acids, amino acids, minerals, enzymes, and a wide variety of polyphenolic compounds. These confer strong antioxidant and antimicrobial activities, which explain its traditional use in wound healing and food preservation [23]. Although rarely tested in fish nutrition due to its high sugar content, honey could have technological applications in aquaculture, particularly as a natural preservative for seafood products.

**Beeswax** is secreted by worker bees from specialised abdominal glands and is primarily composed of long-chain esters, hydrocarbons, and free fatty acids. It has negligible nutritional value, as it is practically indigestible for vertebrates, but possesses remarkable physical properties as a hydrophobic barrier. Beeswax is widely used in the food industry as a coating to reduce oxidative spoilage and microbial growth, and similar applications could be envisaged in aquaculture for product preservation and storage stability [23,26].

Another emerging bee-derived product is **drone brood** (immature male honeybee larvae), recently investigated as a novel functional food. Although no experimental evidence is yet available in aquaculture, drone brood is rich in proteins, essential amino acids, minerals, and steroid hormones, suggesting potential applications in fish nutrition. Nutraceutical studies have shown that lyophilised drone brood combined with eggshell-derived calcium improved the in vitro bio accessibility of testosterone, oestradiol, calcium, and phenolic compounds [46]. Food technology trials also indicated a favourable mineral profile but highlighted safety concerns related to lead contamination, while lyophilisation was reported to enhance palatability and consumer acceptance [47].

### 5.2. Experimental Evidence in Aquaculture

Although the scientific literature on bee bread, royal jelly, honey, and beeswax in aquaculture is still scarce, some preliminary studies provide insights into their possible roles. The available data are fragmentary and mostly derived from exploratory trials, yet they underline the potential of these products as functional additives.

No controlled feeding trials with **bee bread** have yet been reported in fish or crustaceans. Most assumptions regarding its potential come from studies on pollen, where fermentation is expected to enhance nutrient bioavailability and probiotic effects. In African catfish, dietary inclusion of bee pollen (10–30 g kg^−1^ diet) for 45 days significantly improved growth, intestinal villus morphology, and lactic acid bacteria counts, with the best performance observed at 1% supplementation [21]. In rainbow trout, dietary chestnut bee pollen supplementation (10–40 g kg^−1^ diet) for 60 days significantly improved growth performance, antioxidant status, and immune response, increasing survival against *Aeromonas salmonicida* infection [18]. In meagre, experimental diets containing chestnut bee pollen at (10–40 g kg^−1^ diet) for 12 weeks caused a linear reduction in growth performance and nutrient digestibility, accompanied by intestinal inflammation and increased hepatic stress markers at the highest inclusion level [22]. These findings suggest that bee bread, with its higher digestibility compared to raw pollen, could yield more consistent benefits, but its direct evaluation in aquaculture species is still missing.

**Royal jelly** has been tested in aquaculture only in limited experimental contexts. A zebrafish trial that included RJ at increasing dietary levels (1–64 g kg^−1^ diet, 60 days) revealed significant modifications in lipid metabolism, with an increase in EPA/DHA ratios but also a reduction in total DHA and n-3 PUFA content [25]. These results indicate potential nutritional modulation, though with possible risks to essential fatty acid balance. Beyond zebrafish, no systematic studies on food fish species have been reported. Nevertheless, the known antimicrobial, antioxidant, and immunomodulatory properties of RJ justify further investigation into its role in growth, reproduction, and immunity of cultured species.

**Honey** has rarely been used as a direct dietary supplement in aquaculture, likely due to its high sugar content and risk of metabolic imbalance, especially in carnivorous fish. Most available data concern its application as a post-harvest preservative. Honey-based coatings have been shown to delay oxidative processes and inhibit microbial growth in seafood products, extending shelf-life and preserving sensory quality [23]. Its use in vivo remains largely unexplored, but controlled supplementation at low levels might represent a future avenue for enhancing immunity and energy availability.

No direct evidence of **beeswax** supplementation in aquaculture feeds exists. Owing to its indigestibility, beeswax has no nutritional role, but its functional applications are of technological interest. In the food sector, beeswax is widely used as a natural biodegradable coating, able to protect products from oxidative spoilage and microbial contamination [23,26]. Similar applications could be envisaged in aquaculture, particularly for the preservation and transport of fish products, or as a carrier for bioactive compounds in encapsulated feed formulations.

### 5.3. Effects on Fish Growth, Immunity and Stress Response

The evidence available for minor bee products in aquaculture is very limited and fragmented, but some patterns can be outlined when considering their potential impact on fish physiology.

**Bee bread**. Although direct feeding trials are not available, bee bread is expected to have higher digestibility and stronger functional properties than raw pollen due to fermentation.

Extrapolations from pollen studies indicate that bee-derived fermented products could enhance growth, antioxidant defences, and gut health in omnivorous species such as tilapia and African catfish. In Nile tilapia, dietary honeybee pollen (10–40 g kg^−1^ diet) for 7–30 days improved growth and immunity, providing up to 93% survival against *Aeromonas hydrophila* infection [19]. In African catfish, supplementation (10–30 g kg^−1^) for 21 days enhanced growth, intestinal morphology, and beneficial microbiota [21]. In salmonids, chestnut bee pollen (10–40 g kg^−1^) for 60 days improved growth, antioxidant status, and resistance to *Aeromonas salmonicida* [18]. Conversely, in meagre, inclusion of bee pollen at 10–40 g kg^−1^ for 90 days reduced growth performance and induced intestinal stress and inflammation at the highest inclusion level [22]. These contrasting outcomes suggest that bee bread could be beneficial in omnivorous species but requires caution in carnivorous fish.

**Royal jelly**. The only aquaculture evidence initially came from zebrafish trials, where RJ supplementation altered lipid metabolism, increasing EPA/DHA ratios but reducing total DHA and n-3 PUFA content [25].

Beyond this, further studies have expanded the evidence base. In zebrafish, dietary supplementation at 1–64 g kg^−1^ diet for 60 days enhanced growth and modulated growth/immune gene expression (best performance around 1.6%, with IGF-1 upregulation at 6.4%) [29]; under crowding stress, 100 g kg^−1^ diet for 30 days improved intestinal antioxidant capacity and antioxidant gene expression, though without rescuing growth at high density [28]; Over the long term, 50–100 g kg^−1^ diet for 74 days fed increased body weight (notably in females), maintained liver health, and improved serum lipid profile [30].

In zebrafish, long-term dietary supplementation with royal jelly (5–10% of diet, 72 weeks) significantly improved growth, reproductive performance, and blood lipid profile, enhancing hepatic function without evidence of toxicity [30].

**Honey**. Once considered marginal in aquaculture, honey is now being evaluated as a dietary prebiotic. In common carp, dietary inclusion of Kapok flower honey at 5–10 g kg^−1^ of feed for 30 days significantly improved growth performance, feed conversion ratio, intestinal microvilli density, and amylase activity, with the best results observed at 10 g kg^−1^ [32]. In African catfish, supplementation with 100 g kg^−1^ of honey for 84 days (equivalent to 0.1 mL g^−1^ feed) produced the highest survival rate (≈95%) and growth indices, whereas higher levels reduced performance [31]. Similar effects have been reported in Nile tilapia, where controlled honey supplementation at 2.5–10 g kg^−1^ of diet for about 30 days enhanced growth, digestive enzyme activity, and intestinal villus morphology, with optimal outcomes around 10 g kg^−1^ [23]. Collectively, these results confirm that honey can act as a natural prebiotic and growth promoter in aquaculture, in addition to its established role as a natural preservative for seafood products. However, its high sugar content and potential metabolic implications—particularly in carnivorous species—should be carefully evaluated in long-term feeding trials.

**Beeswax**. Beeswax has no nutritional function in fish and no direct evidence of use in aquaculture diets exists. However, as a hydrophobic and biodegradable material, it can act as a coating or encapsulating agent for bioactives, potentially protecting feeds or fish products from oxidation and microbial degradation [23,26]. Its effects on growth or immunity remain speculative and purely indirect.

Comparative overview. Overall, minor bee products show species- and dose-dependent outcomes, with beneficial effects more evident in omnivorous species (e.g., tilapia, catfish, zebrafish) and potential adverse responses in carnivorous fish (e.g., meagre). The recent dietary trials with honey and royal jelly demonstrate that these products are not only of technological interest but can also directly influence growth, immunity, and metabolism. By contrast, bee bread and beeswax remain underexplored, with most assumptions drawn from related products or technological applications. Taken together, these findings strengthen the rationale for systematic evaluation of all minor bee products in aquaculture through controlled feeding studies.

### 5.4. Limitations and Research Needs

The use of bee bread, royal jelly, honey, and beeswax in aquaculture is constrained by several major limitations. The most evident is the scarcity of experimental studies. With the exception of a handful of trials on royal jelly and honey—such as zebrafish studies on oligosaccharide supplementation [28,29,32], tilapia fed honey-supplemented diets [31], and channel catfish trials with royal jelly [30]—no controlled feeding experiments have been reported for bee bread or beeswax. For honey and beeswax, additional knowledge still derives mostly from their application in food preservation rather than direct nutritional use [23,26].

A second critical limitation is the high variability in composition. Bee bread quality depends on both pollen origin and fermentation-associated microbiota, while royal jelly varies in protein and 10-HDA content according to colony status, bee genetics, and collection stage. Honey composition is influenced by floral sources and processing, and beeswax quality is linked to hive conditions. Such variability complicates standardisation and the establishment of reproducible dosages for aquaculture use.

A third challenge is the risk of adverse effects or inefficacy. The meagre trial with pollen supplementation showed impaired growth and intestinal alterations at higher doses, underscoring the possibility that products with complex matrices may negatively affect carnivorous marine fish [22]. Similarly, the high sugar content of honey and the lipid-rich composition of royal jelly could disturb metabolic balance if not carefully integrated into formulated feeds. Beeswax, being indigestible, has no nutritional value and its relevance remains restricted to technological uses.

From a methodological perspective, most available data are short-term and laboratory-based, with limited endpoints. Long-term trials, evaluation of reproductive performance, stress resilience, and product quality are lacking. Challenge tests with pathogens and contaminants—already applied in pollen and propolis studies [13,17]—are virtually absent for minor bee products.

Future research should focus on:

1. Designing controlled dose–response studies in both omnivorous and carnivorous fish species.
2. Implementing standardised chemical characterisation of bee bread, royal jelly, honey, and beeswax before use in diets.
3. Expanding functional endpoints to include growth, immunity, oxidative stress, gut health, reproduction, and product quality.
4. Conducting long-term and farm-scale validation to assess sustainability and economic feasibility.
5. Exploring technological applications, such as coatings or encapsulation systems, where honey and beeswax may provide added value.
6. Investigating emerging bee-derived products such as drone brood, which have been characterised for their nutritional value and functional properties in human nutrition but remain completely untested in aquaculture; trials are needed to assess digestibility, safety, and bioactivity in fish species before any practical application can be recommended [46,47].

In conclusion, while the evidence for bee bread and beeswax remains largely speculative, recent dietary trials with honey and royal jelly provide encouraging results in zebrafish, tilapia, and channel catfish. These findings highlight that minor bee products warrant further systematic investigation. Their cautious integration into aquaculture nutrition and product management could contribute to sustainable and functional innovations in the sector.

**Figure 5:**
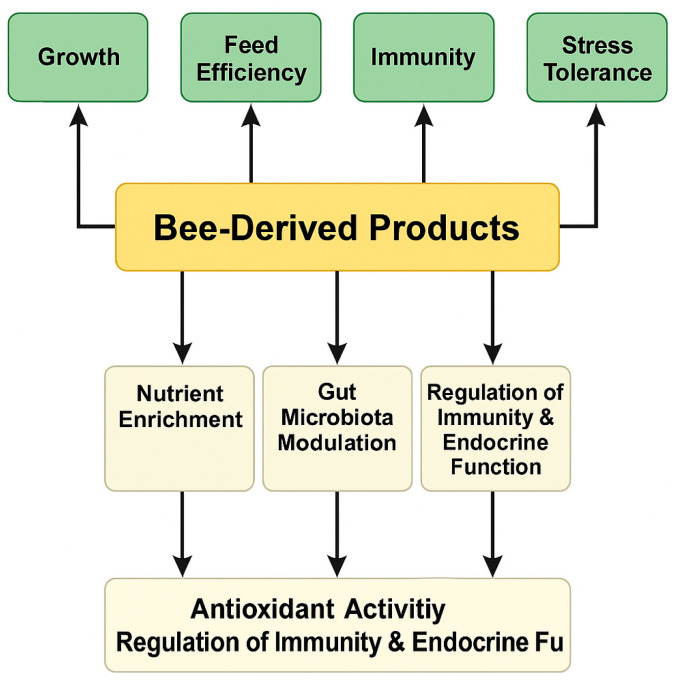
Summary of the main physiological mechanisms and outcomes associated with bee-derived product supplementation in aquaculture.

To visually summarise the relationships discussed above, Figure 5 presents a conceptual overview of how bee-derived products influence fish physiology under aquaculture conditions. The diagram highlights the main mechanistic pathways—including nutrient enrichment, modulation of gut microbiota, and regulation of immune and endocrine functions—that collectively enhance growth performance, feed efficiency, immunity, and stress tolerance. This visual synthesis supports the experimental evidence presented in the following section and facilitates understanding of the multifactorial actions of bee products in aquatic species.

## 6. Product Quality and Consumer Perception

Beyond their effects on growth performance and immunity, bee-derived products may also influence the quality of aquaculture products. Several studies have demonstrated that dietary supplementation with bee pollen or propolis can modulate lipid metabolism in fish muscle, favouring a greater deposition of polyunsaturated fatty acids (PUFAs) and improved oxidative stability during storage [7,19]. Such improvements are particularly relevant for enhancing the nutritional value of fish fillets, since a higher PUFA content is associated with recognised human health benefits. In addition, the antioxidant and antimicrobial compounds of bee products have been considered for use in natural coatings or preservatives, delaying lipid peroxidation and microbial spoilage, thereby extending shelf-life and maintaining sensory quality [23].

Nonetheless, some limitations emerge depending on species and product type. In carnivorous fish such as meagre, excessive levels of bee pollen negatively affected growth, nutrient digestibility and intestinal morphology, with implications for product quality [22]. Conversely, positive effects on gut microbiota and intestinal histomorphology have been observed in African catfish, suggesting potential benefits for nutrient absorption and overall quality of fish flesh [21]. These findings indicate that the use of apicultural additives in aquaculture not only supports fish health during farming but also contributes to producing products of higher nutritional and commercial value, consistent with consumer demand for natural and sustainable innovations in aquaculture.

### 6.1. Nutritional Profile

Bee-derived products not only affect fish growth and immunity but can also improve the nutritional quality of aquaculture products. Supplementation with bee pollen or propolis has been associated with enhanced lipid metabolism in fish muscle, resulting in a higher deposition of polyunsaturated fatty acids (PUFAs) [7,19]. These effects are partly explained by the antioxidant compounds naturally present in these products, such as flavonoids and phenolic acids, which stimulate endogenous enzymatic defences including superoxide dismutase (SOD), catalase (CAT), and glutathione peroxidase (GPx). As a result, lipid peroxidation is reduced, as indicated by lower malondialdehyde (MDA) levels, while omega-3 fatty acids are better preserved.

In addition to lipids, bee products protect muscle proteins from oxidative damage, limiting carbonylation and preserving texture and water-holding capacity of fillets. Some studies also suggest positive effects on digestive enzyme activity, such as amylase and protease, which enhance nutrient utilisation and contribute to a more favourable muscle composition [2,7]. Overall, these mechanisms indicate that bee-derived products enrich the nutritional profile of aquaculture products and improve their stability over time.

### 6.2. Shelf-Life and Sensory Quality

The bioactive molecules of bee products also play a key role in prolonging shelf-life and preserving sensory properties. Phenolic compounds in propolis and honey exert bacteriostatic and bactericidal effects against spoilage microorganisms, while their antioxidant activity limits the oxidative degradation of lipids and proteins. These mechanisms reduce rancidity, preserve colour, and maintain freshness and desirable flavour for longer periods.

Recent reviews highlight the potential of incorporating bee-derived extracts into edible coatings and natural preservatives, which extend product stability without the use of synthetic additives [23]. This “clean label” approach is particularly valuable in aquaculture, where microbiological safety and sensory quality are crucial for consumer acceptance.

Beyond conventional physicochemical and sensory parameters, the use of electronic sensing systems—including electronic nose (e-nose), electronic tongue (e-tongue), and electronic eye (e-eye) technologies—offers new perspectives for the objective evaluation of fish product quality. These instruments, already widely used in food science and particularly in the assessment of freshness, colour stability, flavour profile, and spoilage dynamics of fish and seafood, can detect subtle variations in volatile, non-volatile, and visual attributes that may reflect diet-related influences. Although no studies have yet applied these technologies to fish derived from aquaculture trials supplemented with bee-derived products, their integration could represent a promising advancement for future research. This approach would help elucidate how bee-related bioactive compounds may modulate sensory properties and consumer perception of aquaculture products.

In salmonids, flesh colour strongly influences market value. A controlled trial in rainbow trout using oil-extracted bee pollen carotenoids (25–50 mg/kg diet, 60 days) produced measurable pigmentation (SalmoFan^TM^ ≈ 20–21) and increased fillet carotenoid content versus a non-pigmented control (TCC: 0.50–0.60 vs. 0.31 mg kg^−1^), although synthetic astaxanthin at 50 mg/kg yielded higher redness (a*) and SalmoFan^TM^ (≈25.7) and the greatest TCC (1.62 mg kg^−1^). This “green extraction” strategy avoids toxic solvents and can be operationalised via pellet oil-coating, offering a natural alternative that may meet consumer preferences, while recognising its lower pigmenting efficacy at the tested doses [26].

In line with this, experimental trials in gilthead seabream demonstrated that dietary supplementation with a 10 g kg^−1^ diet supercritical fluid extract of bee pollen (HBP_SFE) did not negatively affect fillet colour, texture or water-holding capacity, nor did it mitigate lipid oxidation over 110 days of frozen storage at −20 °C, indicating that storage time rather than diet was the main driver of quality changes [34].

### 6.3. Consumer Acceptance

Consumer perception is increasingly shaped by expectations of natural and sustainable food production. The use of bee-derived products in aquaculture fits this trend, as they represent multifunctional additives that improve fish health while also enhancing product quality. However, acceptance is closely tied to species-specific responses and consistent product outcomes.

In carnivorous species such as meagre, high inclusion levels of bee pollen impaired growth, digestibility, and intestinal morphology, potentially lowering product quality and consumer appeal [22]. Conversely, in African catfish, bee pollen supplementation improved intestinal histomorphology and promoted beneficial microbial populations, enhancing nutrient absorption and supporting better flesh quality [21]. Although direct evidence of short-chain fatty acid (SCFA) production is lacking, the promotion of lactic acid bacteria suggests a prebiotic potential that could contribute to improved intestinal health and product quality.

From a market perspective, aquaculture products enriched or preserved with natural antioxidants are aligned with consumer expectations of healthier and more sustainable foods. Clear communication of these benefits through transparent labelling could enhance consumer trust and justify premium pricing. Moreover, the integration of apiculture and aquaculture aligns with circular economy principles, offering opportunities for added value and sustainability across food systems.

Overall, the evidence indicates that bee-derived products can positively influence the nutritional profile, shelf-life, and market value of aquaculture products, although species-specific differences and dosage effects remain critical factors. By enhancing oxidative stability, supporting favourable lipid composition, and offering natural preservative functions, these products provide a dual benefit for both producers and consumers. Nevertheless, the variability of outcomes observed across species highlights the need for standardised protocols and further validation at industrial scale. These considerations set the stage for a broader critical analysis of the current literature and the identification of key knowledge gaps, which will be addressed in the following section.

## 7. Critical Considerations and Research Perspectives

One of the main obstacles to the integration of bee-derived products into aquaculture is their high intrinsic variability. The chemical composition of pollen, propolis, and honey is strongly influenced by botanical origin, geographical source, seasonality, and extraction or processing methods, resulting in heterogeneous outcomes that make it difficult to establish uniform recommendations [26,45]. In the case of pollen and bee bread, the diversity of bioactive constituents associated with different floral sources further contributes to inconsistent biological responses [4,5].

This variability is compounded by the absence of standardised protocols for dosage, formulation, and administration: studies differ considerably in the forms tested—from crude powders and ethanolic extracts to microencapsulated preparations or edible coatings—as well as in inclusion levels, trial duration, and experimental conditions, making comparisons difficult and hindering the identification of optimal dosages [7,19,20,43,44].

Despite the encouraging evidence collected, the marked heterogeneity among fish species, experimental designs, product formulations, and outcome measures still limits the possibility of drawing robust, generalisable conclusions. To overcome these limitations, future investigations should adopt more structured synthesis frameworks—such as systematic reviews or meta-analyses—to quantitatively evaluate the strength of current evidence and to establish more reliable, species- and product-specific recommendations.

Another critical issue concerns the limited coverage of species: most studies have been conducted on freshwater fish such as Nile tilapia and rainbow trout, while data on high-value marine species and crustaceans remain scarce and fragmented, despite some available evidence on seabream, seabass, and meagre [8,21,22,38].

A further limitation is that most available trials are short-term and conducted under laboratory or pilot-scale conditions: although useful for clarifying mechanisms of action, such research fails to fully capture the complexity of commercial aquaculture systems. Large-scale validations, long-term monitoring, and cost–benefit analyses are still lacking, which are fundamental for practical adoption [2,25,45].

Finally, safety and regulatory aspects require greater attention: only a few studies have assessed potential interactions with environmental contaminants or long-term biochemical responses, and systematic data on residues or interactions with veterinary drugs are scarce, underscoring the need for clearer regulatory frameworks and risk assessment guidelines before widespread adoption [3,12,17,45].

### 7.1. Practical Guidelines for Safe Inclusion and Application

From a practical standpoint, the current body of evidence allows a preliminary identification of safe and effective inclusion ranges and processing strategies for different bee-derived products. In most controlled trials, dietary levels of 1–3% for ethanolic or crude propolis extracts and 10–30 g kg^−1^ for bee pollen appear to be well tolerated in freshwater fish, supporting improvements in growth, immunity, and oxidative balance [8,13,14,18,21,22,37,38]. Ethanolic extraction generally provides more stable and bioavailable compounds than crude propolis, while fermentation—as in bee bread—enhances nutrient digestibility and probiotic potential [4,5,7,9,25,26].

In several studies, combinations of bee pollen with propolis or royal jelly have produced additive or synergistic effects on growth performance, antioxidant status, and gut health, suggesting complementary mechanisms among these matrices [10,11,19,20,23].

These evidence-based indications may serve as preliminary guidance for future feed formulation and experimental design, helping to bridge the gap between experimental findings and practical application in aquaculture [2,12,24,27,45].

### 7.2. Opportunities and Research Perspectives

Despite these challenges, bee-derived products offer considerable opportunities for innovation in aquaculture. Advances in analytical methodologies, including high-performance chromatography, mass spectrometry, melissopalynology, and artificial sensing technologies (e-nose, e-tongue), provide powerful tools for improving characterisation, standardisation, and traceability, thereby reducing the variability that currently hampers reproducibility [48,49,50,51]. Future research should expand beyond tilapia and trout to include high-value marine fish and crustaceans, supported by long-term, large-scale industrial trials that can provide decisive evidence for practical application [8,22,38].

From an applied perspective, bee products are especially promising because of their dual functionality: as feed additives that improve growth, immunity, and stress resistance, and as natural preservatives capable of enhancing product quality and extending shelf life [23,24]. Their integration is aligned with clean-label strategies and consumer preferences for sustainable, natural solutions, thus supporting not only animal performance but also the commercial value and acceptance of aquaculture products. Moreover, valorising apicultural by-products within aquaculture systems fits the principles of the circular economy, reducing waste and creating synergies between two complementary sectors. In this context, research should not only focus on biological efficacy but also address regulatory frameworks, safety assessment, and transparent labelling to facilitate market trust and consumer awareness.

## 8. Conclusions and Future Directions

Bee-derived products have emerged as multifunctional additives with the capacity to enhance fish growth, immunity, stress resistance, and product quality, while offering natural alternatives to synthetic compounds. The evidence reviewed here demonstrates their potential to improve both aquaculture performance and consumer-oriented outcomes such as nutritional value, sensory attributes, and shelf-life.

However, the most relevant contribution of these findings lies in the broader perspective: bee products should be considered not as isolated supplements but as strategic resources that integrate animal health, food quality, and sustainability within aquaculture systems. Their valorisation fits well within circular economy principles, linking apiculture and aquaculture in a synergistic way and promoting resource efficiency.

Future research should prioritise three key directions: (i) the expansion of studies to high-value marine fish and crustaceans, moving beyond the current focus on tilapia and trout; (ii) the validation of outcomes through long-term, large-scale industrial trials, including cost–benefit analyses; and (iii) the development of safety assessments, regulatory frameworks, and transparent labelling strategies to facilitate market acceptance.

In particular, controlled feeding trials in high-value marine species and crustaceans are strongly needed to verify the transferability of the observed benefits under saline conditions and to define optimal formulations and dosages for different production systems. Such efforts would help bridge the current evidence gap between freshwater and marine aquaculture and enhance the practical applicability of bee-derived products in diverse farming contexts.

Moreover, future multi-omics approaches (including transcriptomics, proteomics, and metabolomics) could provide deeper mechanistic insights into how bee-derived bioactives modulate immune, oxidative, and metabolic pathways, thereby resolving current inconsistencies among experimental studies and strengthening the overall evidence base.

Achieving these goals will be crucial to translating promising experimental evidence into practical solutions.

In conclusion, bee-derived products represent not only an opportunity for innovation in aquaculture nutrition but also a pathway towards a more resilient, sustainable, and consumer-responsive aquaculture sector, aligned with global food security objectives.

## Figures and Tables

**Table 1:** Bee-derived products: main bioactive compounds, biological activities, and evidence in aquaculture.

Bee-Derived Product	Main Bioactive Compounds	Fish Species (Stage)	Dose (g kg^−1^ Diet)/ Duration (Days)	Main Effects	References
Propolis	Flavonoids, phenolic acids, terpenoids, waxes, essential oils	Nile tilapia (juveniles)	5–30/28–30	↑ growth, ↑ feed efficiency, ↑ lysozyme and serum bactericidal activity, ↑ resistance to *Aeromonas hydrophila*	[13]
		Nile tilapia (juveniles)	5–30/30	variable response; >20 g kg^−1^ reduced performance	[16]
		Nile tilapia (juveniles), BPA exposure) *	9/42	mitigated bisphenol-A toxicity; ↑ growth, ↑ SOD & CAT, ↓ MDA	[3]
		Nile tilapia (juveniles)	10–15/28	↑ growth, improved water quality, optimum = 10 g kg^−1^	[15]
		Rainbow trout (juveniles)	0.5–1.5/56	↑ growth, ↑ n-3 PUFA deposition, ↑ immune response	[7]
Bee Pollen	Proteins, amino acids, fatty acids, vitamins, carotenoids, flavonoids	Nile tilapia (juveniles)	10/60	↑ growth, ↑ immunity, ↑ antioxidant enzymes	[19]
		Rainbow trout (juveniles)	10–40/14	↑ growth, ↑ haematology and antioxidant status, ↑ survival vs. *Aeromonas salmonicida*	[18,26]
		African catfish (juveniles)	10–30/21	↑ growth (+45%), ↑ LAB microbiota, ↑ villus height	[21]
		Meagre (juveniles)	10–40/60	↓ growth, ↓ digestibility, intestinal stress (TNF-α ↑, HSP70 ↑)	[22]
Bee Bread	Fermented pollen enriched with lactic acid bacteria, vitamins, polyphenols	Rainbow trout (juveniles)	10–20/28	↑ digestibility, ↑ intestinal villi length, ↑ antioxidant capacity	[4]
Royal Jelly	Major royal jelly proteins (MRJPs), peptides, fatty acids (10-HDA), vitamins	American catfish (*Rhamdia quelen*) (juveniles, waterborne model) *	10 mg L^−1^/30	mitigated tebuconazole toxicity; ↑ SOD, CAT, GST; ↓ TBARS	[27]
		Zebra fish (*Danio rerio*), Chanel catfish (*Ictalurus punctatus*) (adults/juveniles) *	1–5/30–45	↑ growth, ↑ immunity (↑ IgM), ↓ lipids	[28,29,30]
Honey	Sugars, organic acids, flavonoids, enzymes, phenolic acids	American catfish (juveniles, waterborne) *	10 mg L^−1^/30	↓ oxidative damage from tebuconazole; ↑ SOD, CAT, GST	[27]
		Nile tilapia (juveniles), Carp (*Cyprinus carpio*), Danio rerio (juveniles)	2.5–10/30–60	↑ growth, ↑ feed utilisation, ↑ gut microbiota	[23,31,32]
Bee Venom	Melittin, apamin, phospholipase A_2_, peptides	– (no fish trials yet)	–	Potential immunomodulatory and cytotoxic activities inferred from mammalian models	[23]

Notes: ↑ = increase; ↓ = decrease; PUFA = polyunsaturated fatty acid; SOD = superoxide dismutase; CAT = catalase; GST = glutathione S-transferase; MDA = malondialdehyde; LAB = lactic acid bacteria; TNF-α = tumour necrosis factor α; HSP70 = heat shock protein 70; BPA = bisphenol A (endocrine-disrupting chemical). Waterborne models are indicated with *.

**Table 2:** Main experimental evidence on bee pollen supplementation in fish species.

Species	Dose/Form	Duration	Physiological Parameters	Main Outcomes	References
Nile tilapia	10–30 g kg^−1^ diet of bee pollen	30–60 days	Growth, feed efficiency, villus morphology, microbiota	↑ growth and feed efficiency; improved intestinal morphology; ↑ LAB and beneficial taxa	[20,21]
	10–40 g kg^−1^ diet of raw pollen	10–30 days	Growth, immunity (phagocytosis, lysozyme, NBT), resistance to *Aeromonas hydrophila*	↑ growth and immunity; best survival at 2.5% for 20–30 days	[19]
	25 g kg^−1^ diet of raw pollen	30 days (dimethoate challenge)	SOD, CAT, GPx, MDA, serum biomarkers	↑ antioxidant enzymes; ↓ MDA; protection of liver and gill tissues under toxic stress	[37]
Rainbow trout	10–30 mL/kg hydroalcoholic pollen extract (HPE)	60 days	Growth, feed efficiency, IgM, lysozyme, antioxidant enzymes	↑ growth and protein efficiency; ↑ IgM; ↑ antioxidant enzymes; ↓ cortisol	[7]
	10–40 g kg^−1^ diet of chestnut bee pollen	60 days	Growth, haematology, IgM, survival after *Aeromonas salmonicida*	↑ growth and immune markers; ↑ survival (RPS)	[18]
	Pigmented vegetable oil (PVO) from bee pollen; top-coated pellets at 25 or 50 mg total carotenoids/kg diet	60 days	Growth (periodical), VSI, fillet colour (L*, a*, b*, SalmoFan^TM^), fillet TCC	↔ overall growth; P2 ↑ periodical WG & SGR; VSI ↓ in P1; ↑ fillet pigmentation and TCC vs. control; lower pigmenting efficacy than synthetic astaxanthin.	[26]
African catfish	10–30 g kg^−1^ diet of bee pollen	21 days	Growth, gut histology, microbiota	↑ growth (45%); improved intestinal villi; ↑ LAB	[21]
Meagre	20–40 g kg^−1^ diet of bee pollen	90 days	Growth, digestibility, intestinal histology, stress markers	↓ growth and digestibility; intestinal damage; ↑ TNF-α and HSP70 at 4%	[22]
Gilthead seabream; Nile tilapia	5–10 g kg^−1^ diet of SFE pollen combined with probiotics	60 days	Growth, immunity, muscle quality	No growth effect; ↑ peroxidase, protease/antiprotease, bactericidal activity; ↑ antioxidant enzymes; improved fillet traits	[33]
Gilthead seabream	50–100 g kg^−1^ diet of crude pollen; 5–10 g kg^−1^ diet of SFE extract	30 days	Growth, hepatic cytokines (*il-1β*, *il-6*, *il-8*), fillet colour, texture, WHC, TBARS	1% SFE ↑ *il-1β*, *il-6*, *il-8*; crude pollen ↓ *il-8*; effect on fillet quality or oxidation n.s.	[34]
Zebrafish	3% of body weight per meal, up to two meals per day)	60 days	Vertical immunity transfer, larval survival	↑ larval survival after bacterial/viral challenge; maternal effect	[35]
	10–30 g kg^−1^ diet of pollen powder	30–60 days	Gut microbiota, tumour progression	Beneficial microbiota modulation; ↑ tumour growth in melanoma-bearing fish	[36]

Notes: ↑ increase; ↓ decrease; ↔ no change vs. control; n.s. = not significant; LAB = lactic acid bacteria; RPS = relative percent survival; SFE = supercritical fluid extract; SalmoFan^TM^ = colour score scale for salmonid pigmentation; TCC = total carotenoid content; WHC = water-holding capacity; TBARS = thiobarbituric acid reactive substances (lipid oxidation index); *il-1β*, *il-6*, *il-8* = interleukins 1β, 6 and 8; IgM = immunoglobulin M; VSI = visceral somatic index; WG = weight gain.

**Table 3:** Main experimental evidence on propolis supplementation in fish species.

Species	Dose/Form	Duration	Physiological Parameters	Main Outcomes	References
Nile tilapia	10 g kg^−1^ diet of crude propolis or ethanolic extract (EE)	30 days	Growth, lysozyme, bactericidal activity, neutrophils, resistance to *Aeromonas hydrophila*	↑ growth and immunity; ↑ survival	[13,16]
	9 g kg^−1^ diet of ethanolic extract	45 days (with BPA exposure)	SOD, CAT, GPx, MDA, AST, ALT, ALP, liver/gill histology	↓ oxidative stress; normalised biochemical profile; hepatoprotection	[3]
	10–15 g kg^−1^ diet of ethanolic extract	30 days	Growth, morphometry, water quality interactions	Best growth at 10 g/kg; effects linked to salinity and conductivity	[15]
	0–4 g kg^−1^ diet of ethanolic extract	2 trials (postlarvae, juveniles)	Growth, condition factor, intestinal histology	Neutral on performance; ↑ condition factor; ↑ protein deposition; intestines unaffected	[43]
Nile tilapia juveniles	0.5–1.5 g kg^−1^ diet of ethanolic extract	45 days s	Digestibility, intestinal histology	↑ energy digestibility (dose-dependent); improved villus morphology at ~1%; growth n.s.	[43]
Mozambique tilapia (*Oreochromis mossambicus*)	10–20 g kg^−1^ diet of ethanolic extract	67 days	Growth, lysozyme, phagocytosis	↑ growth and feed efficiency; ↑ innate immune responses	[44]
Rainbow trout	0.5–9 g kg^−1^ diet of propolis	60 days	Serum biochemistry, growth	Safe up to 9 g/kg; no adverse effects	[17]
	Brown propolis extract at 1–3 g kg^−1^ diet	30 days	Growth performance	Optimal growth at 2.2 g/kg	[14]
European sea bass	1.25–2.5 g kg^−1^ diet of crude propolis powder	70 days + cold stress test	Growth, RNA/DNA ratio, biochemical parameters under acute cold stress	↑ SGR, FCE, ALP; ↓ triglycerides; improved tolerance to cold stress	[8]
	4.5 g kg^−1^ diet of propolis	56 days + 18 days at 32 °C (heatwave)	Growth, survival, RBC, Hb, lysozyme, cortisol, *hsp70*, antioxidant enzymes	↑ growth & survival; ↑ RBC, Hb, lysozyme; ↓ cortisol & *hsp70*; improved antioxidant status	[39]
	4.5 g kg^−1^ diet of propolis	45 days + 20 days at 7 °C (cold stress)	Cortisol, glucose, lactate, hepatic enzymes, gene expression (*srebp1*, *fads2*, *glut2*)	↓ cortisol & lactate; stable glucose; ↑ *srebp1*; preserved *fads2* & *glut2*; no effect on growth pre-stress	[40]
	4.5 g kg^−1^ diet of propolis	56 days + 18 days at 32 °C (heatwave)	Cortisol, hepatic/renal enzymes, *igf1*, *Tnf-α*, *glut2*	↓ cortisol & enzyme leakage; ↑ *igf1*, *tnf-α*, *glut2*; improved metabolic resilience	[41]
	4.5 g kg^−1^ diet of propolis at salinity 12 vs. 30 PSU	60 days + 20 days at 30 °C (heatwave)	Growth, cortisol, SOD, GPx, *hsp70*, *igf1*, *fads2*, *Tnf-α* (tissue-specific)	12 PSU + propolis → ↑ growth, SOD, ↓ cortisol; 30 PSU + propolis → ↑ *hsp70*, *igf1*, *fads2*, *tnf-α* in liver; enhanced stress resilience	[42]
Gilthead seabream	Ethanolic extract (in vivo)	Variable	Innate immunity (respiratory burst, phagocytosis, lysozyme)	↑ innate immunity; protection against pathogens	[38]
Nile tilapia	Ethanolic extract at 5–30 g kg^−1^ diet	30 days	Growth, haematology, immunity, resistance to *Aeromonas. hydrophila*	Immunostimulation; inconsistent effects on growth	[16]

Notes: ↑ increase; ↓ decrease; →leads to; n.s. = not significant; PSU = practical salinity unit (measure of seawater salinity); SGR = specific growth rate; FCR = feed conversion ratio; FCE = feed conversion efficiency; RNA/DNA ratio = growth/metabolic index; RBC = red blood cells; Hb = haemoglobin; ALP = alkaline phosphatase; AST = aspartate aminotransferase; ALT = alanine aminotransferase; CAT = catalase; GPx = glutathione peroxidase; GST = glutathione S-transferase; MDA = malondialdehyde; SOD = superoxide dismutase; *igf1* = insulin-like growth factor 1; *tnf-α* = tumour necrosis factor alpha; *hsp70* = heat shock protein 70; *srebp1* = sterol regulatory element-binding protein 1; *fads2* = fatty acid desaturase 2; *glut2* = glucose transporter 2; EEP = ethanolic extract of propolis; BPA = bisphenol A.

## Data Availability

No new data were created or analysed in this study.
